# The importance of peripheral vision when searching 3D real-world scenes: A gaze-contingent study in virtual reality

**DOI:** 10.1167/jov.21.7.3

**Published:** 2021-07-12

**Authors:** Erwan Joël David, Julia Beitner, Melissa Le-Hoa Võ

**Affiliations:** 1 Department of Psychology, Goethe-Universität, Frankfurt, Germany

**Keywords:** visual search, visual fields, visual attention, gaze-contingent protocol, virtual reality, scene grammar

## Abstract

Visual search in natural scenes is a complex task relying on peripheral vision to detect potential targets and central vision to verify them. The segregation of the visual fields has been particularly established by on-screen experiments. We conducted a gaze-contingent experiment in virtual reality in order to test how the perceived roles of central and peripheral visions translated to more natural settings. The use of everyday scenes in virtual reality allowed us to study visual attention by implementing a fairly ecological protocol that cannot be implemented in the real world. Central or peripheral vision was masked during visual search, with target objects selected according to scene semantic rules. Analyzing the resulting search behavior, we found that target objects that were not spatially constrained to a probable location within the scene impacted search measures negatively. Our results diverge from on-screen studies in that search performances were only slightly affected by central vision loss. In particular, a central mask did not impact verification times when the target was grammatically constrained to an anchor object. Our findings demonstrates that the role of central vision (up to 6 degrees of eccentricities) in identifying objects in natural scenes seems to be minor, while the role of peripheral preprocessing of targets in immersive real-world searches may have been underestimated by on-screen experiments.

## Introduction

Navigating our visual environment and accomplishing a task relies on many cognitive processes starting with visual processing of information via the retina. Visual perception across the retina is not uniform; it is best at its center, the fovea, and decreases as a function of eccentricity (Loschky et al., 2005; Rosenholtz, 2016). This effect is evident as observed through photoreceptor density (Curcio et al., 1990; Oyster, 1999) and receptive field size (Dacey, 1993; Croner & Kaplan, 1995; Nassi & Callaway, 2009). Cortical representation of peripheral information is compressed farther, giving more weight to central vision (Schira et al., 2007, 2010). As a result, peripheral vision is not precise but accounts for a much bigger surface on the retina compared to central vision; as such, it is geared toward building a quick but coarse global representation of the environment (Larson et al., 2009; Loschky et al., 2019; Trouilloud et al., 2020). It is sufficient to reliably achieve visual processing tasks such as object and scene perception (Boucart et al., 2013, 2016; Thibaut et al., 2016; Loschky et al., 2019), color perception (Na et al., 2006; Hansen et al., 2009), or action recognition (Fademrecht et al., 2016) at high retinal eccentricities (50 to 70 degrees).

Functionally, peripheral vision serves to explore a scene, while central vision is used to sequentially analyze regions of interest with high resolution (Nuthmann, 2014). Attentional guidance in a scene is determined by peripheral contextual information helping prioritize the analysis of regions relevant to a given task (Neider & Zelinsky, 2006; Boucart et al., 2013; Pereira & Castelhano, 2014; Wu et al., 2014). Nuthmann (2013) measured the visual span of on-screen visual search to be approximately 8 degrees of field of view (centered on the fovea). According to this study, low-pass filtering the peripheral content of the field of view beyond 8 degrees has no significant influence on visual search performances. In another on-screen study, Nuthmann (2014) reports that the first stages of visual search (building an initial representation of the scene and scanning for a target) are not impaired by the masking of foveal and parafoveal vision (up to 4.1 degrees). Therefore, at least when performing two-dimensional (2D), on screen searches in natural scenes, the use of peripheral vision is limited to below 8 degrees of eccentricity to the fovea. The small size on the visual field of stimuli presented on screen may explain Nuthmann's results. In virtual reality (VR) conditions where the excited field of view is significantly larger and objects are bigger, these findings may not stand.

When searching for objects in real-world scenes, scene grammar (Võ et al., 2019; Võ, 2021) may contribute to directing attention toward regions containing potential search targets. For one, objects in real scenes assume a restricted set of possible rotations and positions (scene syntax; Biederman et al., 1982; Võ & Henderson, 2009). Scene semantics, on the other hand, refer to object-to-scene (see Võ & Henderson, 2009) and object-to-object relationships (e.g., co-occurrence, spatial proximity; see Biederman et al., 1982). That is, we would expect a toothbrush to be present in a bathroom or to find a mug next to a tea kettle. Observers are managing their expectations of where to find an object using internalized scene grammar rules (Biederman et al., 1982; Võ & Henderson, 2009; Hwang et al., 2011; Draschkow & Võ, 2017; Võ et al., 2019). Hwang et al. (2011) demonstrated that gaze more often than not moved between objects with semantic proximity and was directed preferentially toward an object semantically similar to a target object. Moreover, observers internalize rules of co-occurrences between objects already from a very young age on (Öhlschläger et al., 2020). Knowing that some objects are often observed at close proximity helps to direct attention (Wolfe et al., 2011; Wu et al., 2014; Võ & Henderson, 2009, 2011; Mack & Eckstein, 2011; Draschkow & Võ, 2017; Boettcher et al., 2018). It follows that smaller local objects (e.g., pots, toothbrush, alarm clock) often gravitate around so-called anchor objects (also known as global objects, e.g., stove, sink, bed). The anchor serves to orient visual attention toward regions where a local, smaller object can probably be found (Boettcher et al., 2018; Võ et al., 2019). Tying it all together: Initial scene gist processing at scene onset provides contextual information from which a map of probable objects and their location can be deduced rapidly, influencing attention scanpaths (Castelhano & Henderson, 2007; Võ & Henderson, 2010). Interestingly, an experiment by Võ and Henderson (2009) in which they manipulated syntactic and semantic object congruency in natural scenes during visual search showed effects on central vision processing through an increase in average fixation durations but no evidence of peripheral processing of incongruent objects (see also Võ & Henderson, 2011).

Most of the aforementioned studies were carried out on 2D screens. The bulk of the searches we perform on a daily basis, however, take place in three-dimensional (3D) environments in which we move around and interact with objects. Therefore, it is important to understand how information processed centrally versus in the visual periphery affects real-world search behaviors. Investigations on real-world search behaviors have been accomplished with mobile eye tracking before (Mack & Eckstein, 2011; Howard et al., 2011; Foulsham et al., 2014; Sippel et al., 2014; Keane et al., 2014; Draschkow & Võ, 2016). The present study aims at measuring the role of central versus peripheral vision in VR settings approaching more realistic conditions while keeping a high degree of experimental control. The advantages of using VR in our case are the ease to process data and the possibility to implement gaze-contingent masks to restrict perception to the central or peripheral field of view.

Looking for abstract stimuli among distractors in an image is not the same as looking for an object in a photograph of a room (Wolfe, 2010; Eckstein, 2011; Wolfe et al., 2011). Similarly, we ask if the latter, looking for an object on screen, is akin to looking for an object in a real-world scene while being present in that scene (physically or virtually). Recent findings indicate that guiding factors and strategies during search do not widely differ between on-screen and virtual settings (e.g., Kit et al., 2014; Li et al., 2016; David et al., 2020; Beitner et al., 2021). This seems unexpected, since there are a number of differences between these two types of experimental scenarios. First and foremost, one can use one's whole body to explore a 3D scene on top of eye movements, while in 2D scenarios, the observer's head is usually constrained by a chinrest. In addition, the degree of peripheral vision greatly differs: In contrast to on-screen studies, which stimulate a limited portion of the field of view (≈30 × 20 degrees, e.g., Nuthmann, 2013; Cajar et al., 2016; David et al., 2019), modern VR devices stimulate far more of peripheral vision (≈90 degrees, e.g., Kit et al., 2014; Li et al., 2016; David et al., 2020). Moreover, displaying natural scenes as images on a screen most often makes their elements smaller than they would appear when being in the scene. Being inside a scene makes it omnidirectional (i.e., the scene content is not constrained to the field of view). Instead, just as in real life, an observer may turn around and walk in search of a target. A 3D-modeled scene and a graphical projection per eye (viewport) allow for stereoscopic vision and more realistic object features such as in-scene depth and multiple angles of inspection for one object. Additionally, 3D-modeled objects can vary according to their material's properties (Pharr et al., 2016), which provides important new features to accomplish the task (e.g., transparency, metallicity, glossiness, Toscani et al., 2019, 2020). All of these points add to the naturalness of the subjective experience and give credit to the claim that we implemented a quasi-natural protocol of visual search. In this context, findings related to central and peripheral vision measured on screen probably differ when transitioning to a large field of view because peripheral vision extends much farther outside of the macula. It is unlikely that findings about the role of central and peripheral vision for search would dramatically change; rather, measures regarding the degree to which each field of view is necessary for visual search would probably vary. For starters, the measured visual span during search (Nuthmann, 2013) is suspected to be larger because natural scenes presented on screen take up little of the field of view, and the objects they contain appear smaller on the retina than they would if one was actually present in the scenes. For the same reasons, the portion of central vision that one can do without during visual search (Nuthmann, 2014) might increase. Being able to use a larger peripheral field of view might also speed up finding and redirecting attention to a target object.

Employing modern VR devices combined with mobile eye tracking allows researchers to study visual attention in quasi-natural situations. First, a VR gaze-contingent protocol has technical disadvantages compared to on-screen studies: The eye-movement-to-display-update latency increases slightly, stimuli resolution is lowered, and eye-tracking accuracy can be reduced. But, advancing to VR resolves several shortcomings of on-screen gaze-contingent protocols: The portion of the field of view excited is significantly increased, masking can be applied independently per eye, and neck and body movements are unrestricted. Retrofitting of an eye tracker system in a VR headset allows one to implement protocols that would not be possible with mobile eye-tracking devices.

Being able to use a body's full range of motion and in particular the head to complement the eyes while viewing and exploring scenes is essential in the real world. The head can be compensatory and stabilize the resulting gaze by movements contrary to the gaze (vestibulo-ocular response, Collewijn & Smeets, 2000; Einh et al., 2007; opto-kynetic response, Robinson, 1981; Leigh & Zee, 2015). In synergistic motions, the head makes it possible to prepare saccades targeting anywhere in the field of view and even outside of it (see the “practical field of fixation”; Rötth, 1925; Von Noorden & Campos, 2002). Head movements are even observed accompanying small eye rotations (Ritzmann, 1875; Bizzi et al., 1972; Einh et al., 2007; Hu et al., 2017). Previous research has shown that the role of the head can be as critical as the eyes (Lee, 1999; Doshi & Trivedi, 2012). Recent research suggests that head and eye movements might operate under different control strategies (Anderson et al., 2020; Jacobs et al., 2020). In particular, the head seems to have a tendency toward large shifts of the field of view to explore a scene while eye guidance tends to be directed to visible information.

Gaze-contingent protocols have been extensively used on screen to investigate central and peripheral vision as a way to display different information at once on the two fields of view (Loschky & McConkie, 2002; Foulsham et al., 2011; Laubrock & Cajar, 2013; Nuthmann, 2013, 2014; Nuthmann & Malcolm, 2016; Cajar et al., 2016; David et al., 2019, 2020). They were first used to study reading (McConkie & Rayner, 1975; Rayner & Bertera, 1979). By implementing such a protocol in VR, we manipulated visual input in three different conditions: (a) in a central-masking condition, we removed all information in a 6-degree radius centered on the gaze; (b) in a peripheral-masking condition, the inverse mask was produced by leaving information only in that 6-degree circle; and lastly, (c) a control condition was added where visual information was not manipulated. Target objects in the virtual scenes were chosen in a way that either facilitated or impeded visual search performance. That is, grammatically constrained objects were objects that had a clear anchor (e.g., toothbrush on a sink), whereas unconstrained items showed no clear anchor that could direct gaze and help in the task (e.g., gong in a bathroom; Boettcher et al., 2018; Võ et al., 2019). We report on visual search behaviors and compare our findings to previous on-screen studies.

In a first step, we sought to test participant adaptation to the protocol (virtual environment, task and the gaze-contingent protocol), and we expected average visual search times to decrease as a function of trial blocks. We expected vision loss to impact search time, search initiation time, scanning time, verification time,^1^ scanpath length, and scanpath ratio, reflecting difficulties in accomplishing the task without central information to analyze regions of interest or peripheral information to identify potential targets to fixate next (Loschky & McConkie, 2002; Nuthmann, 2013, 2014; Nuthmann & Malcolm, 2016). We analyzed scanpath length in addition to scanpath ratio, because we decomposed it into gaze, eye, and head scanpath length in order to study the contribution of head movements to visual search. We expected head movements to contribute by extending the field of fixation (Rötth, 1925; Von Noorden & Campos, 2002; Einh et al., 2007; Freedman, 2008). We expected search performances to be high overall but to be reduced in the presence of a mask compared to a no-mask condition (Nuthmann, 2014; Pereira & Castelhano, 2014; Cajar et al., 2020). Previous research has shown little effect of central loss of vision on the first phase of visual search (Nuthmann, 2014; Nuthmann & Malcolm, 2016). Thus, we expected initiation time not to be affected by central masking. Nuthmann (2014) and Nuthmann and Malcolm (2016) reported no significant increase of average scanning times with a central mask. However, choices of mask radius in these two on-screen studies were smaller than in the present study (maximum of 3.5 and 4.1 degrees, respectively). In our case, we expected a mask of 6 degrees of radius to have an equivalent effect as in these previous studies when accounting for the size of the field of view and of objects in the scene. In contrast, we expected average verification times to increase when central vision was masked as participants cannot use central vision to identify targets efficiently (Nuthmann, 2014; Nuthmann & Malcolm, 2016; Cajar et al., 2020). Since the verification phase requires fine object identification with central vision, we expected peripheral masking not to increase average verification times. We expected target refixation rates to increase with central masking but not peripheral masking, due to an increase in return saccades as participants try to analyze regions of interest despite the gaze-contingent mask (Henderson et al., 1997; Nuthmann, 2014; David et al., 2019). Apart from such global effects on search performance, we hypothesized that some objects within a scene would be affected more than others. For instance, objects with a clear anchor relation would be easier to find than targets missing this extra source of guidance (Boettcher et al., 2018; Võ et al., 2019). Such anchor objects can be identified peripherally and help guide gaze toward potential locations targets that cluster around the anchor (for a review, see Võ, 2021). When only central vision is available, finding an anchor object can lead an observer to explore the region around that anchor object preferentially in hope of finding the target. Effects of peripheral loss of vision should be increased when searching for grammatically unconstrained items, apart from verification times and target refixation rates, because the lack of scene grammatical constraint is characterized by impeded guidance in a clustered scene but is less detrimental on identification (Neider & Zelinsky, 2006; Mack & Eckstein, 2011). Once an item is found and foveated, its identification is trivial with a peripheral mask, because their size in the field of view will almost always fit within the gaze-contingent mask. In case of central loss of vision, lack of grammatical constraint should hardly affect average initiation and scanning times but should impact target identification.

## Method

The method of this study is identical to a previous article that focused on the analysis of visuomotor variables (for a detailed description of the method, see David et al., 2020), while here we investigated the effects of central versus peripheral masking on visual search behavior as a function of grammatical constraint. The study plan and analyses were preregistered on the Open Science Framework website.^2^

### Participants

Thirty-two fluent German speakers participated in this experiment (24 women, mean age: 24 years old, minimum: 18, maximum: 43). We intended to recruit 45 participants, but data collection was stopped due to COVID-19. We asserted normal visual perception and the dominant eye before the experiment. All participants gave their written consent before beginning the experiment and were compensated for their time with university credit or 8 €/h. The experiment took a maximum of 60 min, conformed to the Declaration of Helsinki, and was approved by the local ethics committee of the Faculty of Psychology and Sport Sciences at Goethe University Frankfurt.

### Apparatus

Virtual scenes were displayed inside an HTC Vive (HTC, Valve Corporation) VR head-mounted display. The headset's displays are refreshed at 90 Hz and shows a field of view of approximately 90 by 90 degree, binocularly. The headset was retrofitted with a Tobii eye tracker (Tobii Technology), tracking gaze binocularly at 120 Hz with a precision below 1.1 degrees within a 20-degree window centered in the viewports. We estimated the maximum (“worst-case scenario”) latency to be below 30 ms.

Importantly, in this setup, the mask is presented in viewport space, not world (3D) space. It can therefore only lag with regard to the position of one eye in the viewport, not in relation to the combined head and eye movement in world space. During large saccades, however, the head often participates more and is usually accompanied by a short eye movement in the viewport (Malinov et al., 2000; Einh et al., 2007; Freedman, 2008). This results in a rather small mask displacement during saccades and reduced mask positional lag, which should minimize the impact of latency.

### Stimuli

Participants searched for objects in 16 virtual scenes of everyday rooms: three living rooms, three bedrooms, three bathrooms, three kitchens, and four offices. The scenes measured 3.8 by 3.5 m and fit inside the physical room where the experiment took place. The proportions of the rooms’ content were similar to real-world scenes; each room was populated with 36 unique objects, 8 anchor objects, and 28 local objects, of which 6 were selected as target objects. For a description of the scenes, please refer to Helbing et al. (2020). Out of the 16 rooms, one living room was set aside for participants to train with and did not appear after the training phase, leaving 15 rooms for the actual task. The use of 3D-modeled scenes and a VR headset entails that the stimuli used in this study are omnidirectional (i.e., they surround the observer in all directions). One cannot perceive the entirety of the scenes at once; changing the content of the viewport via movements of the head is therefore necessary to explore them fully. A video of rotating views of the VR rooms can be found in the supplementary material.

**Figure 1.**
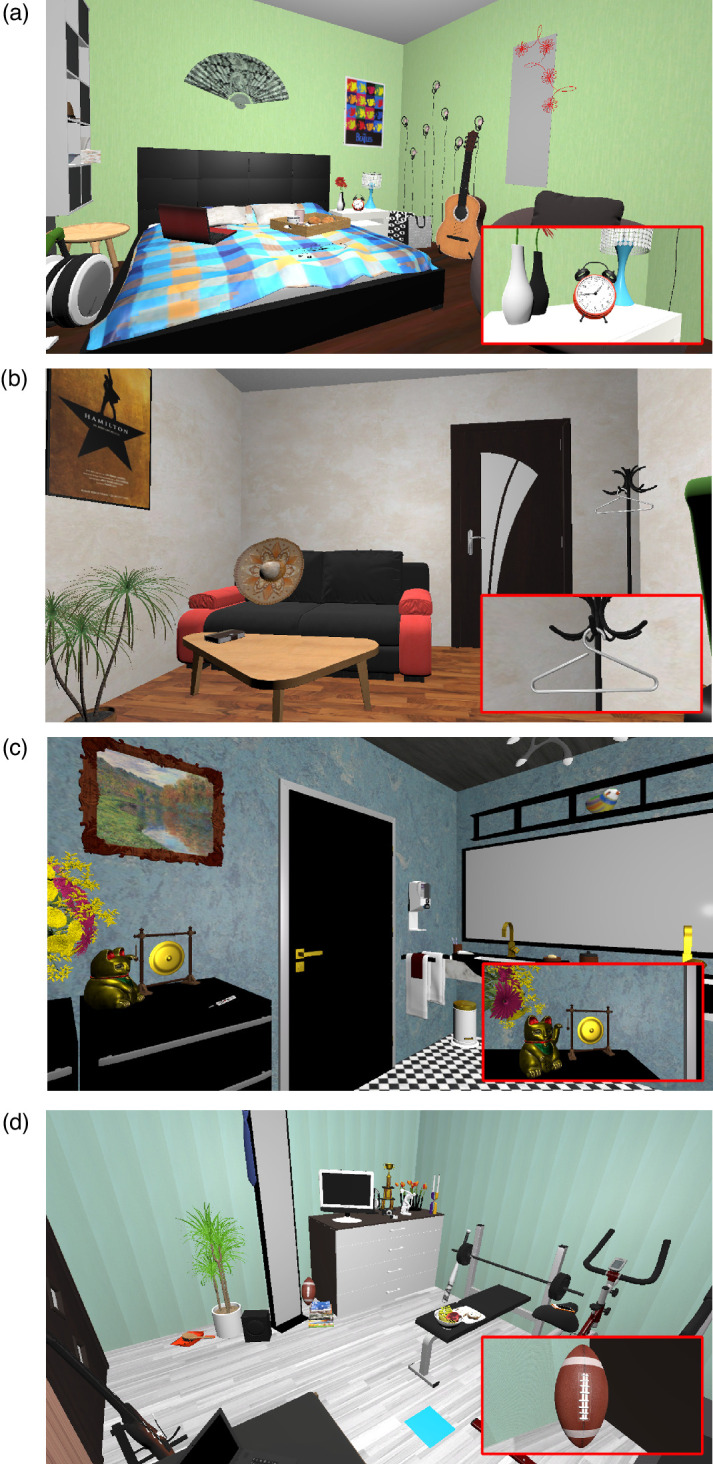
Views from four virtual rooms. The two top images show an example of “grammatically constrained” targets (alarm clock, clothes hanger), which are placed next to objects (bed, coat rack) that are semantically related and anchor objects to these targets. The lower images show two “grammatically unconstrained” targets, located without an anchor object (gong, American football). Zoomed-in views of the target objects are shown within the red rectangles.

A lexicon of German words was built, referencing all objects present in the scenes. Words from this lexicon were used as cues when displaying a target word relating to the object to look for in the scenes. In each scene, six target objects were selected: three grammatically constrained and three unconstrained objects to find (Figure 1). Object grammatical constraint was determined according to “anchorness”: Constrained objects were chosen to have a clear anchor, whereas unconstrained items showed no clear anchor that could direct gaze and help in the task (Boettcher et al., 2018; Võ et al., 2019). The selection was made by visually looking through scenes for objects fitting these definitions.

### Experimental design

**Figure 2.**
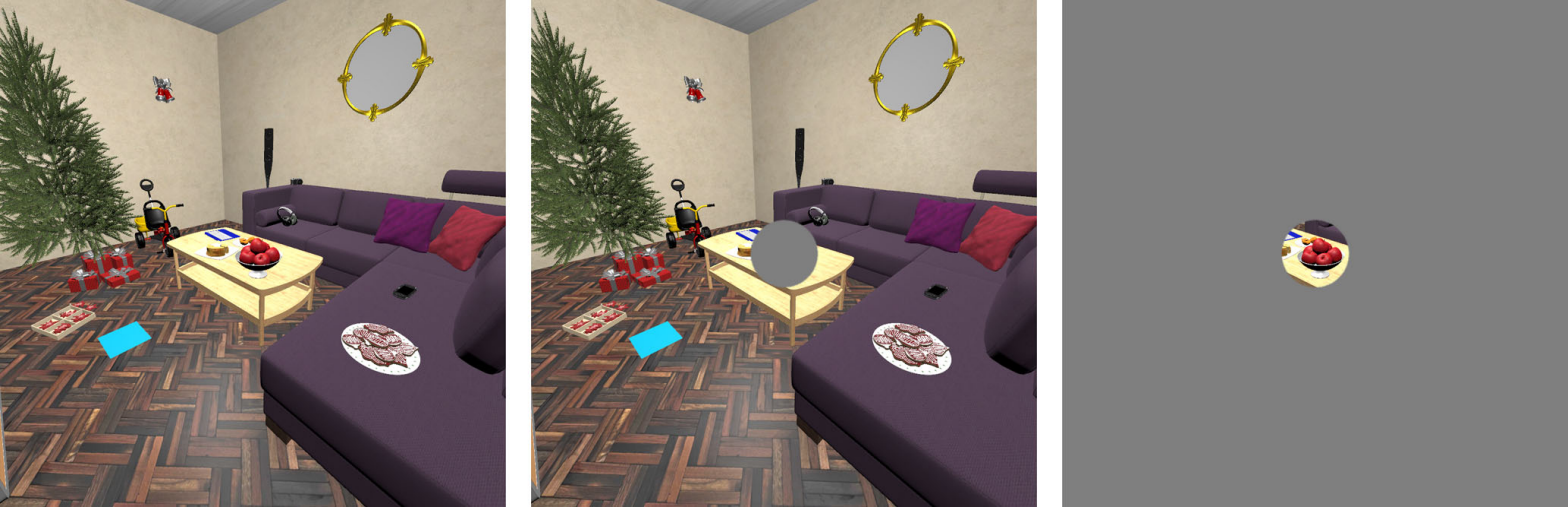
Masking conditions are presented here in a viewport measuring 90 by 90 degrees of field of view; mask radii are proportionally accurate. From left to right: control no-mask condition, central mask of 6 degrees of radius, and peripheral mask of 6 degrees of radius. The captured scene view shows the training room.

We implemented a gaze-contingent protocol in order to remove central or peripheral visual information where a participant's gaze was located in real time (Figure 2). We obstructed part of the scene by adding a gray circular mask centered on gaze positions to obtain central masking. We masked everything but a central circular area to remove information in the peripheral field of view. This method was applied to both eyes: The left viewport was updated with left gaze data and the right one with right gaze data. We chose a circle of 6 degrees of radius for both central- and peripheral- masking conditions on the basis of results from a previous experiment (manuscript in preparation) that showed strong effects of gaze-contingent masking in VR (free-viewing task) with masks of 6 degrees of radius, in terms of eye movement amplitudes and return saccade rates in particular. Moreover, in a pilot study dedicated to testing the implementation of the protocol (six subjects, 540 trials), we implemented central masks of 4, 6, and 8 degrees of radius, as well as peripheral masks measuring 4 and 6 degrees. While both peripheral masks substantially impacted gaze movements, a 6-degree mask was ultimately chosen because the alternative appeared to affect gaze too strongly. On the other hand, results of central masking showed that only the bigger mask (8 degrees) appeared to markedly impact visuomotor variables; nonetheless, we judged that this was removing too much central information and would make comparing results with on-screen studies difficult. Thus, our final choice was of 6 degrees of radius.

In the present study, participants were tasked with finding six target objects in 15 scenes, one at a time, in one of the mask conditions (90 trials plus six training trials). A cued target was always present in the scene.

### Procedure

Participants started the experiment with an eye tracker calibration (9 points) and validation (9 points) procedure. In a training phase, participants got accustomed to the material and the protocol by searching for six objects in a dedicated practice scene that did not appear thereafter. They experimented with masked and control conditions (twice each in a random order). In a pretrial room containing only a black screen on a wall, participants were told to move onto a blue square on the floor (starting position in a scene common to all subjects). They had to fixate the screen for the target cue word to appear for 1 s, before the trial room appeared. The room disappeared after 30 s or after pressing a controller button to register that the target was found. Participants were told to fixate the target as they pressed the controller button. Trials were separated by at least 1 s, a calibration/validation phase was triggered every 10 trials, a resting period was inserted every 30 trials, but participants could take a break after any trial. Participants went through all trials in 40 min on average.

Each participant looked for the same target object only once. We created an experimental “playlist” per participant so that the unique combination of target objects crossed with masking conditions appeared the same number of times across the experiment, considering the preregistered number of subjects. Another constraint of the playlists was that masking conditions had to be balanced across scenes: As a result, across the six target objects, the scene was seen with each masking conditions twice.

### Data preparation

We processed three types of movements: combined head and eye rotations as **gaze** movements (*eye-in-space*), eye rotations as (dominant) **eye** movements (*eye-in-head*), and head rotations as **head** movements (Larsson et al., 2016; Lappi, 2016).

We identified saccades and fixations on the basis of the combined gaze data with a velocity-based algorithm (Salvucci & Goldberg, 2000): We calculated gaze velocity as the orthodromic distance divided by the time difference between two gaze samples. The resulting signal was smoothed with a Savitzky-Golay filter (Nystr & Holmqvist, 2010); filtered samples below 120 deg/ms. were identified as part of a fixation.

Gaze data were saved to file once per frame along with head-tracking information. For each frame, we identified which object in the scene was looked at. After identifying fixations, this determined which object was looked at during a fixation. With this information, we determined verification times as starting with the first fixation on a target and target refixation as the number of times that target was fixated again after that point. A trial was deemed successful if the last fixation was on the target at the time a participant ended the trial.

We removed trials for which more than 15% of either left or right eye-tracking signal was lost (*N* = 77). We removed trials that lasted less than 500 ms. or for which only one fixation was found (*N* = 2). Of the remaining trials, we report 282 unsuccessful trials (10.3%). In the following analyses, all measures but search success rate are based on data from successful trials.

## Analyses

Continuous response variables were analyzed with linear mixed models (Baayen et al., 2008) and binary variables (trial success) with generalized linear mixed models (Jaeger, 2008), in order to account for fixed and random experimental effects. The random effects present in our experiment are subjects, scenes, and objects (present as random intercepts). We tested our hypotheses with planned (dummy) contrasts: the difference between control condition versus central mask and control condition versus peripheral mask (mask condition as a fixed effect), as well as between grammatically constrained and unconstrained targets per masking condition (interaction of mask condition and grammatical constraint as a fixed effect). We consider absolute *t*-values equal or above 1.96 to be significant on the two-tailed 5% level (Baayen et al., 2008; Cajar et al., 2016).

As a complement to the planned contrast comparisons, we estimated main and interaction effects following the method described in Nuthmann and Malcolm (2016).^3^ For each dependent variable, we constructed four models: two including fixed effects alone (mask and constraint models), one including both fixed effects (mask-const. model), and one including the fixed effects and their interaction (full model). Using likelihood ratio tests, three comparisons were run. If the full model had a better fit than the mask model, we interpreted that there was an effect of grammatical constraint (main or interaction); inversely, when comparing the full model to the constraint model, a better fit of the full model was interpreted as there being a main or interaction effect of masking; finally, if the full model showed a better fit than the mask-const. model, then we reported an interaction effect. The result of these comparisons is reported in the appendix (Table A1), along with the analysis of secondary dependent variables.

### Search initiation time

**Figure 3.**
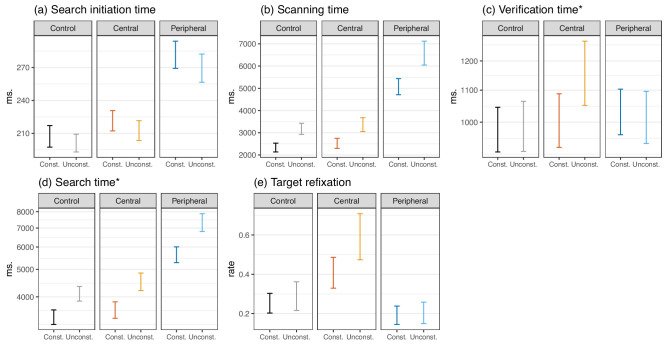
Visual search measures are presented as a function of mask conditions and target object grammatical constraint (mean and 95% CI). On the x-axis are presented object grammatical constraint (“Const.” and “Unconst.”) while mask conditions appear as facets of the subplots. An asterisk to the right of a variable's name indicates that it was log-transformed in linear mixed models and is presented on a log-scale here.

Duration of the first fixation at scene onset accounts for search initiation time. During this short time lapse, a representation (gist) of the scene is constructed that guides visual attention at first (Larson et al., 2009; Loschky et al., 2019). As expected, observers were strongly affected by the removal of peripheral stimulation; on average, the first fixation increased by an estimate of 71 ms in this condition compared to no-mask trials (b=71.01,SE=5.02,t=14.13). Central masking resulted in a smaller effect (b=13.21,SE=5.06,t=2.61). One might conclude that removal of peripheral information had more impact at this stage than central information removal, but it should be noted that the surfaces removed in the two conditions were not equivalent. Thus, while it makes sense that during the initial processing of scene gist, peripheral vision is crucial, such a conclusion based on these two masking conditions is not warranted at this time. Object grammatical constraint did not have a sizable impact on this variable (control: b=-6.33,SE=7.09,t=-0.89; central: b=-8.38,SE=7.66,t=-1.09; peripheral: b=-11.72,SE=7.57,t=-1.55; Figure 3a). At least at this stage, scene grammatical rules do not seem to have an effect.

### Scanning time

Scanning time, measured as the time after the first saccade in the scene and before the first fixation on target, characterizes the degree of guidance to the target during search. Average scanning times during central-mask trials were not different from no-mask trials (b=222.03,SE=156.56,t=1.42). This shows that central vision was not necessary when looking for objects with an extended field of view (Nuthmann, 2014; Cajar et al., 2020). In contrast, peripheral information is critical during this phase (Nuthmann, 2013). In its absence, we observed a substantial increase in the average scanning time estimated at 3,100 ms (b=3099.78,SE=157.13,t=19.73). Object grammatical constraint played a key factor in scanning time, as can be seen during no-mask trials: targets not associated with an anchor took longer to be found in the scenes (b=762.43,SE=217.77,t=3.5). For the two masking conditions, this effect was even more pronounced when peripheral vision was absent (b=1,449.98,SE=258.77,t=5.6) than when central vision was masked (b=757.98,SE=256.86,t=2.95; Figure 3b).

### Verification time

We measured the time it took participants to identify targets as the time between the first fixation on the target and the end of the trial. Average verification times were not affected by the loss of peripheral information compared to control trials (b=0.04,SE=0.04,t=1.2). With a peripheral mask, once gaze latched onto a target object, the usable field of view was large enough to identify them accurately. On the other hand, despite a noticeable variance, there was a main effect of central masking only as seen in longer verification times (b=0.09,SE=0.04,t=2.55). This main effect of central masking seems to be carried by a larger increase in average verification times for grammatically unconstrained compared to constrained objects (b=0.13,SE=0.05,t=2.46; Figure 3c). We ran additional unplanned post hoc comparisons to test if there was an increase in average verification times between the control condition and central masking as a function of target grammatical constraint. Results show that there was no difference between control and central masking for grammatically constrained targets (b=0.02,SE=0.05,t=0.32), but an increase in favor of central masking for unconstrained targets (b=0.17,SE=0.05,t=3.3).

The fact that a central mask did not seem to reliably affect verification times (in particular when searching for grammatically constrained targets) suggests that, at least for predictable targets, visual processing past the parafovea is sufficient to identify targets in natural and complex scenes We infer that peripheral preprocessing was quite effective in accurately identifying targets without having to rely on fixations to verify them. As expected, loss of peripheral information did not show substantial differences on this final search phase as a function of object grammatical constraint (b=-0.02,SE=0.05,t=-0.35), similar to the control condition (b=-0.01,SE=0.05,t=-0.28).

#### Search time

The total amount of time needed to find, identify a target object, and press the controller's trigger to end the trial is identified as search time. A linear mixed model with an intercept per mask condition and a slope for trial blocks (nine) per mask condition shows that average search times decreased with trial block counts. The model estimates that, on average, search times decreased by 3.7% per trial block in the control trials (b=-0.04,SE=0.01,t=-4.55) and 3% in the central-mask condition (b=-0.03,SE=0.01,t=-3.57). The decrease was lower in peripheral, mask trials (1.5%; b=-0.01,SE=0.01,t=-1.73). Thus, search performance improved as participants experienced more trials and constructed representations of the scenes (Howard et al., 2011; Kit et al., 2014). This improvement was not as important when peripheral information was removed.

As expected, average search times were longer during peripheral-mask trials compared to no-mask trials (b=0.55,SE=0.03,t=18.36). Central masking also affected search times, but with much less impact (b=0.08,SE=0.03,t=2.65). Regardless of masking conditions, grammatically unconstrained target objects resulted in longer search times (control: b=0.17,SE=0.04,t=3.98; central: b=0.2,SE=0.05,t=4.45; peripheral: b=0.24,SE=0.05,t=5.24; Figure 3d). This reiterates the advantage of placing objects near anchors and the internalization of object-to-object rules in the sampled participants (Võ et al., 2019).

### Target refixation

The number of fixations falling on a target object after it was once fixated is a measure of uncertainty and difficulty to identify objects. As expected, compared to the no-mask condition, we measured more refixations with a central mask (b=0.23,SE=0.04,t=6.33), whereas peripheral-mask trials were not affected (b=-0.07,SE=0.04,t=-1.96). Previous works showed that a high number of return saccades are produced when central vision is not available as observers make back-and-forth movements between a region of interest and the rest of the scene (Henderson et al., 1997; Cornelissen et al., 2005; David et al., 2019). Target grammatical constraint had no effect on average target refixation rates during no-mask (b=0.02,SE=0.05,t=0.32) and peripheral-mask trials (b=0,SE=0.06,t=-0.04; Figure 3e). An effect of grammatical constraint moderated by a strong variance is observed during central-mask trials (b=0.18,SE=0.06,t=3.23). It seems that the identification uncertainty of a target was decreased when it appeared in proximity of a semantically related anchor. The absence of such information in the case of grammatically unconstrained targets along with the impossibility to foveate them would explain the increase in target refixation rates.

## Discussion

Stimuli used in visual search studies that address the role of central and peripheral vision have increased in complexity in an effort to approach conditions that are natural. The question of ecological experimental validity (Holleman et al., 2020) is important to us because we are studying a visual task that humans do every day in complex environments. With VR devices, we moved further into the direction of more realistic stimuli and search conditions. In this study, we implemented a gaze-contingent protocol in VR and masked either central or peripheral vision during visual search of objects in virtual every day scenes. Use of a head-mounted display increased the naturalness of the gaze-contingent protocol: no head or body restraints, an extended field of view, and masks applied independently per eye. We begin this discussion by comparing our results to past on-screen experiments to answer the question of validity of findings made on screen in relation to real-world behavior. We report on the impact of vision losses on the different phases of visual search and the significance of scene grammar. Our results have implications regarding the role of central and peripheral visions and the visual span of visual search.

### Comparison of our results to on-screen research

As expected, participants most generally managed to find the target within the allotted time, and their performance steadily increased over time, indicating that they adapted to the novelty of the material and the peculiarities of the protocol. As was reported in a 2D on-screen study by Nuthmann (2014), in our experiment, a central mask only slightly impacted scanning and search initiation times. This reflects the ease with which participants built a scene gist at trial onset and navigated the scene by identifying potential targets peripherally. We had therefore expected to observe an effect of peripheral masking on this same measure of time, in accordance to the on-screen literature (Nuthmann, 2013, 2014; Nuthmann & Malcolm, 2016). Search initiation times increased as observers could not build a representation of the scene; scanning times also increased due to the limited spotlight shone on the scene, inadequate to find targets. Verification times only differed from no-mask trials when central vision was removed and the target was not in proximity of a semantically related anchor object (i.e., it was not strongly constrained by scene grammar). Central masking impacted verification times when the target object was not grammatically constrained. We hypothesize that thanks to the anchor-relatedness, those objects were able to be identified more easily in the periphery with a high level of judgment. In the absence of this additional information predicted by the anchor, participants needed more time to arrive at a sufficiently high level of certainty for target identification. We know that peripheral perception is sufficient to process scenes, faces, and objects in the far periphery (Boucart et al., 2013, 2016), but no findings about visual search in a natural environment with an extended field of view had so far been available. We also know that removing 6 degrees centrally impacted visuomotor processes because we observed an increase in average saccade amplitudes and backward saccade rates in a previous study analyzing visuomotor variables of that same experiment (David et al., 2020). We deduce that the visual field beyond the 6 degrees of eccentricity is sufficient to identify objects in more naturalistic conditions to a degree that decreases the role of central vision for making the final target decision. As expected, peripheral masking did not affect verification time (Nuthmann, 2013, 2014). Target refixation rates measures how many times a participant gazed at a target after looking at it for the first time. Refixations can happen because an observer could not inhibit a saccade; continuing along a scanning line (saccadic momentum), a return saccade is then made to verify the target. Alternatively, participants may not notice the target and keep scanning the scene for it, before fixating it later again. Few refixations were measured; participants refixated during one in four peripheral and no-mask trials. This rate almost doubled during central-mask trials. Overall, one look at a target was all it took; we should mention that we explicitly asked participants to look at the object when pressing the controller trigger so that we could identify misfires. It is realistic to believe that participants were capable of identifying targets peripherally but pressed the trigger after making a saccade because of our instructions rather than an actual need to do so.

### The role of central and peripheral vision

The strong effect of transient peripheral vision loss on almost all measures studied reiterates the importance of peripheral vision in visual search to build a representation of the scene in order to detect potential targets. Our main finding is that contrary to results from on-screen search experiments, central masking did not always affect target verification time. For instance, lack of central vision had little impact on search performances, implying a greater role for peripheral preprocessing of targets when searching with an extended field of view. Considering the visual span of visual search, the results of this study allow us to assert that it is larger than 6 degrees: A peripheral mask of this size had a strong impact on initiation and scanning times, and a central mask had only little impact on verification time, especially for grammatically constrained targets. This is in contradiction with on-screen experiments implementing smaller masks on screen (Nuthmann, 2013, 2014). The visual span, or useful field of view, is the portion of the field of view that is necessary to normally achieve a visual task. It can be measured by masking peripheral vision. The corollary to the useful field of view would be to understand how much of central vision can be masked before search performances become significantly reduced; in effect, the visual span would be defined as a portion of the visual field in the shape of a ring with lower and upper radius bounds. In pattern recognition tasks using drawn scenes, the effective visual span was measured to be close to 5 degrees of radius (Saida & Ikeda, 1979; Shioiri & Ikeda, 1989). Nuthmann (2013) presented natural scenes on screen in a gaze-contingent protocol, low-pass filtering peripheral information, and reported the visual span of object search to be approximately 8 degrees. It should be noted again that in the present study, the virtual scenes are omnidirectional; they surround the observer and therefore extend beyond the visual field in all directions; being able to preprocess potential targets using more of the periphery is very important to the task. Considering our results, we believe that, in natural settings, the visual span is probably much larger than so far identified on screen. Future experiments are required to measure the visual span of visual search in more detail in a quasi-natural environment.

### Importance of scene grammar for attentional guidance

In addition to modifying the field of view, we also manipulated object grammatical constraint, in relation to “anchorness,” since they have proven to be important for attentional guidance in real-world scenes (Draschkow & Võ, 2017; Boettcher et al., 2018). We defined object constraint as constrained objects placed near a congruent anchor (e.g., a toothbrush on a sink) and unconstrained objects positioned without an anchor (e.g., a gong in a bathroom). As expected, the lack of grammatical constraint of an object mostly affected search guidance and less so measures of object identification (verification time and target refixations). We report an increase in verification times and target refixation rates with a central mask. Here, we partly replicate on-screen literature showing the impact of central masking on scene guidance, which we show in conjunction with unconstrained targets. In general, the effect sizes observed in masking conditions were similar to those of the no-mask condition. A key feature of anchors is that due to their relatively big size, they can be detected faster peripherally and thus function as an anchoring for predictions about smaller objects. We see this in particular during central-masking trials, where anchor objects could be identified peripherally, thereby guiding gaze toward a grammatically constrained target in its vicinity. Without this additional source of guidance, the boosting of peripheral processing by scene grammar was lost, thus increasing verification times and refixation rates. Moreover, average scanpath ratios and lengths were shorter when a target was constrained to an anchor (see Appendix). Additionally, objects located next to a congruent anchor were identified faster and with fewer refixations when central vision was removed, implying that an anchor increased observers’ confidence about the target when high- acuity data were not available. This result supports the literature showing that semantically inconsistent or syntactically misplaced objects slow down search significantly (Võ & Henderson, 2011; Võ & Wolfe, 2013), which speaks for the importance of grammatical constraint on search behavior.

Insights on how one could make better use of their field of view by utilizing scene grammar cues like anchors could help in finding solutions (assisting tools, coping mechanisms) supporting individuals suffering from visual field losses (e.g., macular degeneration or glaucoma). Our findings indicate that patients affected by central visual field loss may possess enough peripheral information to navigate their environment in order to find every day objects (Tran et al., 2010; Boucart et al., 2013; Thibaut et al., 2014, 2016). In their study, Thibaut et al. (2018) used a 180-degree panoramic screen. People with central visual field defects searched for objects in natural scenes and accomplished the task at accuracy levels only slightly lower than that of an age-matched group. Difficulties appear when handling objects to accomplish everyday tasks (Boucart et al., 2015), as it was shown that central vision more often than not accompanies the locus of action (Land et al., 1999; Hayhoe et al., 2003; Hayhoe & Ballard, 2005). Considering our results, one could propose that people living with central vision losses could be supported by placing objects at expected locations, in order to rely on scene rule priors to assist in everyday life. However, an experiment by Geringswald et al. (2013) showed that patients suffering from central visual field defects did not efficiently learn from contextual cues to improve visual search of simple shapes, like normally sighted participants did. Nevertheless, this does not mean that contextual information learned through lifelike scene grammar could not help. Instead, they might have failed to learn artificial, experimentally controlled regularities in scenes. Moreover, compared to what we learned from on-screen studies, central vision loss may be better compensated in 3D due to more use of peripheral vision.

## Conclusion

VR headsets with embedded eye-tracking are a great opportunity to study visual search in near-natural settings. In this study, we had participants search for objects as they were immersed in complex virtual scenes mimicking everyday rooms. They used the full range of motion of their body as well as a large field of view to accomplish this task. With a gaze-contingent protocol implemented in VR, we were able to replicate most findings reported on on-screen gaze-contingent studies. However, our results diverge in one important aspect: Contrary to our expectations and previous reports from 2D studies, loss of central vision did not strongly impact visual search measures, in particular when looking for grammatically constrained targets (i.e., those that tend to be found next to a congruent anchor object). For those objects, verification times did not differ from control trials. Observers were able to identify targets without central vision at a high accuracy and made use of scene semantic information to identify potential targets faster and increase their confidence in their target identification decision. Our main finding indicates that when immersed in a 3D environment, peripheral information processing is used more reliably to identify search targets than reported by on-screen studies. Our results also imply that the visual span of visual search is probably larger than reported previously using on-screen experiments. We believe that state-of-the-art VR devices with integrated eye trackers are mature enough for scientific purposes, and we encourage the community to make use of VR paradigms to study the role of central versus peripheral vision beyond what has been possible when using traditional screen monitors.

## Supplementary Material

Supplement 1

## Figures and Tables

**Figure A1.**
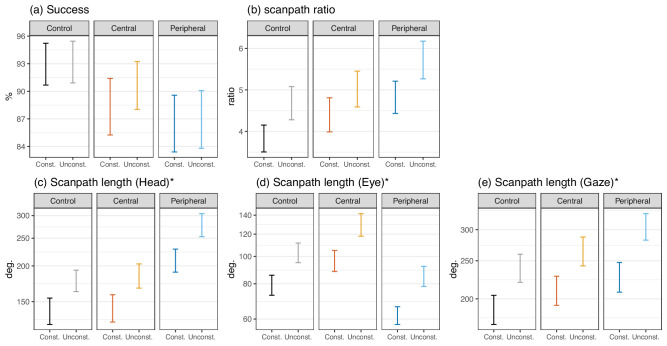
Visual search measures are presented as a function of mask conditions and target object grammatical constraint (mean and 95% CI). On the x-axis are presented object grammatical constraint (“Const.” and “Unconst.”) while mask conditions appear as facets of the subplots. An asterisk to the right of a variable's name indicates that it was log-transformed in linear mixed models and is presented on a log-scale here.

## Footnotes

1 Analysis of “verification times” was not planned in the preregistration document.

2
https://osf.io/5n8ce/.

3 This analysis was not planned in the preregistration document.

## Appendix

### Likelihood ratio tests

**Table A1.** Likelihood ratio tests are reported in this table in order to estimate main and interaction effects associated with measures of search behavior.

Dependent variable	Test	Χ^2^	*Pr*(> Χ^2^)
Search initiation time	Effect of mask	216.47	< 0.0001
	Effect of gram. const.	4.7	0.2
	Interaction effect	0.27	0.87
Scanning time	Effect of mask	438.12	< 0.0001
	Effect of gram. const.	62.14	< 0.0001
	Interaction effect	6.24	0.044
Verification time	Effect of mask	12.36	0.015
	Effect of gram. const.	7.14	0.068
	Interaction effect	5.83	0.054
Search time	Effect of mask	360.77	< 0.0001
	Effect of gram. const.	65.97	< 0.0001
	Interaction effect	1.39	0.5
Target refixation	Effect of mask	79.38	< 0.0001
	Effect of gram. const.	12.32	0.006
	Interaction effect	7.6	0.022

Dependent variable	Test	Χ^2^	*Pr*(> Χ^2^)

Success	Effect of mask	24.43	< 0.0001
	Effect of gram. const.	0.83	0.084
	Interaction effect	0.79	0.675
Scanpath ratio	Effect of mask	29.94	< 0.0001
	Effect of gram. const.	23.25	< 0.0001
	Interaction effect	0.42	0.81
Scanpath length (head)	Effect of mask	116.57	< 0.0001
	Effect of gram. const.	39.39	< 0.0001
	Interaction effect	0.085	0.958
Scanpath length (eye)	Effect of mask	116.46	< 0.0001
	Effect of gram. const.	66.42	< 0.0001
	Interaction effect	0.47	0.79
Scanpath length (gaze)	Effect of mask	35.01	< 0.0001
	Effect of gram. const.	46.03	< 0.0001
	Interaction effect	0.63	0.73

### Analysis of supplementary search measures

#### Success

We measured success rate as the proportion of trials for which a target was correctly identified within the allotted trial duration. We considered a trial to be successful when participants were looking at the target during the second before they pressed the controller trigger. On average, participants successfully identified the target in 89.8% of trials. Generalized linear models (binomial distribution: logit link function) were constructed. As expected, success rates were high overall, and the presence of a gaze-contingent mask provoked a decrease in object search accuracy compared to the no-mask condition (central: b=-0.53,SE=0.18,t=-2.98; peripheral: b=-0.81,SE=0.17,t=-4.78). The lack of grammatical constraint did not impact performances (control: b=-0.07,SE=0.28,t=-0.24; central: b=0.22,SE=0.23,t=0.94; peripheral: b=-0.01,SE=0.21,t=-0.06; Figure A1a).

#### Scanpath ratio

Scanpath ratios measure the total distance traveled by gaze divided by the minimum scanpath length (minimum gaze rotation needed to fixate a target object from the position at the onset of a trial). All target objects were visible from onset positions by rotation alone; therefore, the minimum scanpath length is equal to the angle between a starting gaze position and the vector that originates at the head in the direction of the target object. Without central vision, we observed an increase in average scanpath ratios (b=0.48,SE=0.19,t=2.48); this increase was stronger when peripheral information was missing (b=1.01,SE=0.17,t=5.78). Akin to search time, scanpath ratio testifies to a difficulty of navigating scenes requiring more exploration. Grammatical constraint impacted no-mask (b=0.77,SE=0.27,t=2.88) and peripheral-mask trials (b=0.9,SE=0.29,t=3.07; Figure A1b) in the same manner by increasing overall ratio when targets were not located near a semantically related anchor object. Central masking resulted in a smaller effect (b=0.65,SE=0.3,t=2.2) in comparison.

A low scanpath ratio determines optimal gaze behavior in terms of movements. It compares the total gaze amplitude to an optimal movement (shortest distance between gaze at trial onset to a fixation on the target object). When observers could not rely on peripheral information to build a scene representation and detect potential targets, the scanpath ratio increased by one third compared to control data. This is in line with studies showing a strong effect of peripheral masks and little to no effect of central masks (Nuthmann, 2013, 2014).

#### Scanpath length

Scanpath length is measured in degrees and represents the total distance traveled by head, eyes, or the combined gaze. This is calculated as the sum of orthodromic distances between fixation centroids.

When head and eye movements were combined, only peripheral masking affected scanpath lengths to a large extent (central: b=0.11,SE=0.04,t=2.85; peripheral: b=0.23,SE=0.04,t=5.88). In this study, gaze scanpath length effects followed scanpath ratio results, but masking impacted eye scanpath lengths differently. Average eye movements during saccades were longer with a central mask (b=0.22,SE=0.04,t=5.64) due to the increase in eye movement amplitude during saccades and a higher backward saccades rate. This increase was not reflected on head movements; the average head movement lengths with a central mask were similar to the control trial average (b=0.03,SE=0.04,t=0.67). Despite the increase in scanning time, average eye scanpath lengths were reduced compared to the control condition with a peripheral mask (b=-0.21,SE=0.04,t=-5.44), because of the strong reduction in average eye amplitudes and the increase in average head movement lengths (b=0.43,SE=0.04,t=9.87).

Lack of associated anchor objects near targets increased average scanpath lengths for head (control: b=0.23,SE=0.06,t=3.7; central: b=0.23,SE=0.06,t=3.56; peripheral: b=0.25,SE=0.06,t=3.9; Figure A1c), eye (control: b=0.24,SE=0.05,t=4.35; central: b=0.27,SE=0.06,t=4.87; peripheral: b=0.3,SE=0.06,t=5.31; Figure A1d), and gaze (control: b=0.21,SE=0.05,t=3.84; central: b=0.2,SE=0.06,t=3.55; peripheral: b=0.26,SE=0.06,t=4.59; Figure A1e) and movements. This effect was predictable considering the similar increase in average scanning times reported previously. The placement of grammatically unconstrained targets made participants look toward potential anchors to a target, or in the absence of a clear anchor, they had to scan the entire scene, a “brute-force” strategy that increases overall movement lengths.

The impact of masking on average eye scanpath lengths reflects the increase in eye saccade amplitudes and backward saccade rates: a longer scanpath with a central mask but nonsubstantial changes with a peripheral mask. During central-mask trials, participants moved their eyes more but found the targets much faster than in peripheral trials. This is why the increase in scanpath length was not reflected on gaze scanpath length and ratio.
